# Immunological feedback loops generate parasite persistence thresholds that explain variation in infection duration

**DOI:** 10.1098/rspb.2024.0934

**Published:** 2024-09-25

**Authors:** Clayton E. Cressler, Daniel C. G. Metz, David A. Chang van Oordt, Andrea L. Graham

**Affiliations:** ^1^ School of Biological Sciences, University of Nebraska-Lincoln, Lincoln, NE, USA; ^2^ Department of Ecology & Evolutionary Biology, Princeton University, Princeton, NJ, USA

**Keywords:** infection duration, Allee effect, immunity

## Abstract

Infection duration affects individual host fitness and between-host transmission. Whether an infection is cleared or becomes chronic depends on the complex interaction between host immune responses and parasite growth. Empirical and theoretical studies have suggested that there are critical thresholds of parasite dose that can determine clearance versus chronicity, driven by the ability of the parasite to manipulate host immunity. However, the mammalian immune response is characterized by strong positive and negative feedback loops that could generate duration thresholds even in the absence of direct immunomodulation. Here, we derive and analyse a simple model for the interaction between T-cell subpopulations and parasite growth. We show that whether an infection is cleared or not is very sensitive to the initial immune state, parasite dose and strength of immunological feedbacks. In particular, chronic infections are possible even when parasites provoke a strong and effective immune response and lack any ability to immunomodulate. Our findings indicate that the initial immune state, which often goes unmeasured in empirical studies, is a critical determinant of infection duration. This work also has implications for epidemiological models, as it implies that infection duration will be highly variable among individuals, and dependent on each individual’s infection history.

## 1. Introduction

The duration of an infection (i.e. the time elapsed between establishment and clearance of an infectious agent) is arguably determined by the within-host ecological dynamics of parasite biomass growth and mortality: the infection lasts until the immune response kills or expels the parasite, or until the parasite otherwise dies. These within-host dynamics have profound implications for both individual host fitness and parasite transmission among hosts. At the individual scale, the time it takes to clear an infection can affect the likelihood that the infection will become lethal [1], the cumulative severity of symptoms [2] and the amount of tissue damage that must be repaired if the individual is to recover [3,4]. At the population scale, the longer the individuals are infected, the longer they tend to be infectious to others [5–7]; infection duration (or its inverse, clearance rate) is thus a canonical parameter in epidemiological models [8,9]. Both the mean and variance in infection duration are expected to affect epidemic outbreak risk and ease of control [10].

Yet, determinants of infection duration are poorly understood. Duration arises from a complex, dynamic interplay of host and parasite genetics with the environment. In general, ‘resistant’ host genotypes clear parasites more rapidly than ‘susceptible’ genotypes do, via powerful immune responses that mobilize appropriate effector mechanisms (e.g. secretion of mucus by goblet cells, accelerated epithelial turnover and peristalsis to expel gastrointestinal nematodes [11]). At the same time, parasite genotypes that best immunosuppress (e.g. [12]) or co-opt resources of the host (e.g. [13]) often generate infections of the longest duration. Infection duration thus varies with host genotype [14–16], parasite genotype [17–19] and sometimes both (e.g. G_H_ × G_P_ interactions for duration [20,21]).

Considerable effort has been expended to understand the molecular and cellular mechanisms behind these genetic effects, yet genetics (even G_H_ × G_P_ interactions) is not the only driver of variation. Indeed, infection duration also varies strongly with environmental factors such as doses or rates of exposure [22–24]. For example, ‘resistant’ mouse genotypes are able to quickly clear high doses of the gastrointestinal parasite *Trichuris muris*—thus displaying acute infections—but become chronically infected when the dose is reduced, with an immune profile that is more similar to ‘susceptible’ mouse strains given a high dose [11,24–26]. A key immunological cause of the host genotypic effect is that at high doses, ‘resistant’ genotypes mount a strong T-helper 2 (Th2) immune response whereas ‘susceptible’ genotypes mount an inappropriate T-helper 1 (Th1) response and become chronically infected, presumably until the worms senesce and die. This led researchers to suggest that a threshold dose may be required to initiate a strong Th2 response and rapidly clear infection (with thresholds likely to differ among host strains) [24].

The notion that thresholds govern infection outcomes such as duration is also suggested by experimental infections of fruit flies [27] and flour beetles [28]. In both systems, the duration of infection was acute in some insects, and chronic in others, despite stringent controls, with acute versus chronic outcomes being determined by subtle differences in the baseline immune state and initial rates of immune response induction and parasite replication.

Here, we explore whether the existence of thresholds in duration and the subtle dependence of duration on initial conditions and early events are indicative of Allee effects driven by strong feedback mechanisms in the within-host ‘ecosystem.’ Allee effects are a relatively common phenomenon in ecological systems [29]. They arise when positive feedback loops generate a positive relationship between per-capita growth rate and population density. The key dynamical signatures of Allee effects are persistence thresholds (e.g. [30,31]): when density is below the threshold, the population declines to extinction; above it, the population persists. Near the threshold, subtle differences in the system state can produce strikingly different persistence times. Two recent mathematical models suggest parasite-driven feedback mechanisms that could produce Allee effects. van Leeuwen *et al*. [32] showed that parasites can avoid clearance if they can force hosts to reallocate resources away from immunity and towards parasite growth. Ellner *et al*. [33] showed that parasites can avoid clearance by directly interfering with the immune response by sequestering, inactivating and inhibiting immune effectors. In both models, the key to producing persistence thresholds was that host manipulation was dependent on parasite biomass; that is, there was positive feedback between parasite biomass and parasite per-capita growth rate—more biomass led to more manipulation, which led to faster biomass growth. This positive relationship between parasite per-capita growth rate and parasite biomass is the hallmark of an Allee effect.

We suggest that positive feedback loops, which are ubiquitous within the mammalian immune response, make Allee effects an intrinsic feature of immune dynamics, with critical implications for the duration of infection. Here we derive and analyse a minimal model for the interaction between parasite biomass and induced T-helper immune responses that incorporates key immunological feedbacks, such as the positive feedback between cytokine production and T-cell activation, and mutual inhibition between T-cell subpopulations [34–36]. We show that this model produces thresholds that can determine acute versus chronic infections. Furthermore, we explore how changing the relative strength of different feedback mechanisms alters the possible dynamical outcomes of the system, recapitulating empirical patterns like the dose-dependence of infection outcome. We suggest that building these mechanistic immune feedbacks into models of host–parasite interactions provides testable insights into the within-host determinants of a crucial immunoepidemiological variable: infection duration.

## 2. Results

We begin by considering the interaction between two of the main effector ‘arms’ of the mammalian adaptive immune response: T-helper 1 and T-helper 2 cells (though, in principle, the feedback loops might also describe interactions among additional T-helper subsets including T-regulatory or T-helper 17 cells [37]). Th1 and Th2 cells are primary coordinators of the adaptive immune response against intracellular microparasites and macroparasites, respectively, and polarization towards one cell type or the other drives parasite clearance or chronicity. This baseline model ignores the dynamics of the parasite to determine whether the positive feedbacks intrinsic to the dynamics of immunity are sufficient to generate immunological Allee effects. Subsequent models will then consider a dynamic parasite.

### (a) Immune feedbacks and initial conditions generate multiple stable equilibria

We assume that there is T-cell independent activation of Th1 and Th2 production, at rates governed by the parameters $b_{1}$ and $b_{2}$ . Activated T-helper cells further increase their population sizes by producing the same cytokines that induced their own activation, termed self-activation. At the same time, the cytokines produced by activated T-helper cells prevent naïve T-helper cells from becoming T-helper cells of the opposite type, termed cross-inhibition. In reality, the activation of a naïve T-cell depends on the intracellular interaction between cytokines and the master regulator transcription factors T-bet and GATA3 [35,37]: Th1 cytokines stimulate the expression of T-bet, and T-bet expression regulates the production of Th1 cytokines; Th2 cytokines stimulate the expression of GATA3, and GATA3 expression regulates the production of Th2 cytokines. Interestingly, T-bet and GATA3 expression are *also* self-activating and cross-inhibiting, which has been shown to produce multistability in individual immune cell phenotypes [35–37]. For simplicity, we here assume that both transcription factor expression in individual cells and the production of cytokines can be captured by (i) a self-activation term that is dependent on the density of T-helper cells of the same type, according to a Hill function with an exponent of p , $\frac{s_{i}T_{i}^{p}}{S_{i}^{p}+T_{i}^{p}}$ , where $s_{i}$ is the maximum self-activation and $S_{i}$ is the self-activation at half-maximum and (ii) by a cross-inhibition term that is dependent on the density of T-helper cells of the opposite type, also according to a Hill function with an exponent of q , $\frac{I_{ij}^{q}}{I_{ij}^{q}+T_{j}^{q}}$ , where $I_{ij}$ is the half-saturation constant for cross-inhibition. Finally, we include immune cell apoptosis due to any of a number of processes [34] with the parameter m . The resulting system of two equations is


$$\frac{dT_{1}}{dt}=\underset{\begin{array}{c} External\;\\ activation \end{array}}{\underbrace{b_{1}}}+\underset{Self-activation}{\underbrace{\left(\frac{s_{1}T_{1}^{p}}{S_{1}^{p}+T_{1}^{p}}\right)}}\underset{Cross-inhibition}{\underbrace{\left(\frac{I_{12}^{q}}{I_{12}^{q}+T_{2}^{q}}\right)}}-\underset{Apoptosis}{\underbrace{mT_{1}}}$$

$$\frac{dT_{2}}{dt}=b_{2}+\left(\frac{s_{2}T_{2}^{p}}{S_{2}^{p}+T_{2}^{p}}\right)\left(\;\frac{I_{21}^{q}}{I_{21}^{q}+T_{1}^{q}}\right)-mT_{2}.$$

In the electronic supplementary material, we show that the possible dynamical outcomes of the model are highly sensitive to the choice of Hill exponents, so for the remainder of our analysis we will focus on the case where p=2 and q=1 , which produces switch-like behaviour in immune self-activation and is supported by previous analyses [35]. To analyse the possible dynamics of this system, we study the nullclines for this system: combinations of $T_{1}$ and $T_{2}$ that cause $dT_{1}/dt=0$ ( $T_{1}$ nullcline) or $dT_{2}/dt=0$ ( $T_{2}$ nullcline). Intersections of these nullclines represent the possible equilibria of the system.

Determining parameter values for this system is challenging: although there are good estimates for the parameters of underlying models of transcription factor expression and cytokine production [36], the complex dynamics produced by such models make the use of quasi-equilibrium assumptions infeasible as a method to estimate the parameters of this model. Instead, we will avail ourselves of the technique of non-dimensionalization often favoured in the analysis of these models [33,34,37]; the dimensionless parameters of the model typically have simpler interpretations. Specifically, we define dimensionless state variables $t_{i}=T_{i}/S_{i}$ and τ=tm . Thus, the dimensionless measure of each immune cell population is relative to the total immune cell abundance such that self-activation is half its maximum ( $S_{i}$ ), and time is measured relative to the average lifespan of an immune cell ( 1/m) . We also define the dimensionless parameters $\beta_{i}=b_{i}/mS_{i}$ , the baseline activation of naïve T-cells; $\sigma_{i}=s_{i}/mS_{i}$ , the maximum T-helper cell self-activation and $\iota_{ij}=I_{ij}/S_{j}S_{j}$ , the half-saturation constant for cross-inhibition. See table 1 for expanded interpretations of these parameters. The dimensionless system is then:

.$$\frac{dt_{i}}{d\tau }=\beta_{i}+\left(\frac{\sigma_{i}t_{i}^{2}}{1+t_{i}^{2}}\right)\left(\frac{\iota_{ij}}{\iota_{ij}+t_{j}}\right)-t_{i}\;for\;i,j=1,2$$

**Table 1.** Summary of dimensionless parameters, including their derivation from dimensional parameters, and default values used in most simulations. See figure captions for specific parameter values.

dimensionless parameters	definition	value(s)
$\beta_{i}=\frac{b_{i}}{mS_{i}}$	baseline activation of naïve T-cells — ratio of the baseline immune activation rate and the immune apoptosis rate when the self-activation rate is half its maximum	1.11×10^−4^
$\sigma_{i}=\frac{s_{i}}{mS_{i}}$	maximum T-helper cell self-activation — ratio of the maximum self-activation rate and the immune apoptosis rate when the self-activation rate is half its maximum	2.22
$\iota_{ij}=I_{ij}/S_{i}$	half-saturation constant for cross-inhibition — ratio of the half-saturation constant for cross-inhibition and the half-saturation constant for self-activation	variable
$\chi_{i}=\frac{c_{i}}{mS_{i}}$	parasite-induced immune activation — ratio of the parasite-induced immune activation rate and the immune apoptosis rate when the self-activation rate is half its maximum	$\chi_{1}$ = 0.11, 0.14, 0.16; $\chi_{2}$ = 0.11, 0.08, 0.06
$\kappa_{i}=\frac{C_{i}}{K_{p}}$	half-saturation constant for parasite-induced immune activation — ratio of half-saturation constant for parasite-induced immune activation and parasite carrying capacity	0.167
ξ=r/m	parasite maximum growth — ratio of the maximum parasite growth rate and the immune apoptosis rate	3.33
$\alpha =aS_{2}/m$	parasite mortality per immune cell — ratio of the parasite killing rate when the self-activation rate is half its maximum and the immune apoptosis rate	3.33

**Figure 1.**
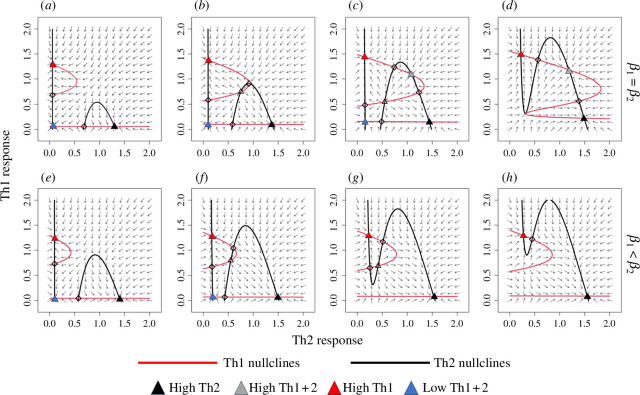
Phase portraits for the dimensionless immune model (equation 2.1) as immune stimulation increases across the columns. Across the top row, $\beta_{1}=\beta_{2}=0.05\;\left(a\right),\;0.08\;\left(b\right),\;0.11\;\left(c\right)\;and\;0.14\;\left(d\right)$ ; across the bottom row, $\beta_{2}=2\beta_{1}$ , with $\beta_{1}=0.04\;\left(e\right),\;0.06\;\left(f\right),\;0.07\;\left(g\right)\;and\;0.075\;\left(h\right)$ . Nullclines are shown by black and red lines. Nullcline intersections represent equilibria. Stable nodes are indicated by filled-in triangles, with the colour indicating the immunological state (as in figure 2); unstable nodes are indicated by hollow triangles; unstable saddles are indicated by hollow diamonds.

Figure 1 shows the configuration of the nullclines as immune activation ( $\beta_{i}$ ) is increased, for example, by the growth of immune-stimulating parasite biomass. The top row of figure 1 shows the nullcline configurations for the case where the Th1 and Th2 responses are balanced (all parameters are equal); the bottom row shows the nullclines when the system is biased towards a Th2 response ( $\beta_{2}>\beta_{1}$ ). In a perfectly balanced system (figure 1, top row), at low immune activation levels, there are three equilibria corresponding to a low activation state and each polarized response, with the basin of attraction being the largest for the low activation state. As activation increases, we see the emergence of many new equilibria, including a stable equilibrium representing high co-activation of both immune response ‘arms’. Eventually, we lose the low activation equilibrium altogether, and further increases in activation would eventually result in the loss of both polarized responses as well, leaving the high coactivation equilibrium as the only possible outcome.

**Figure 2.**
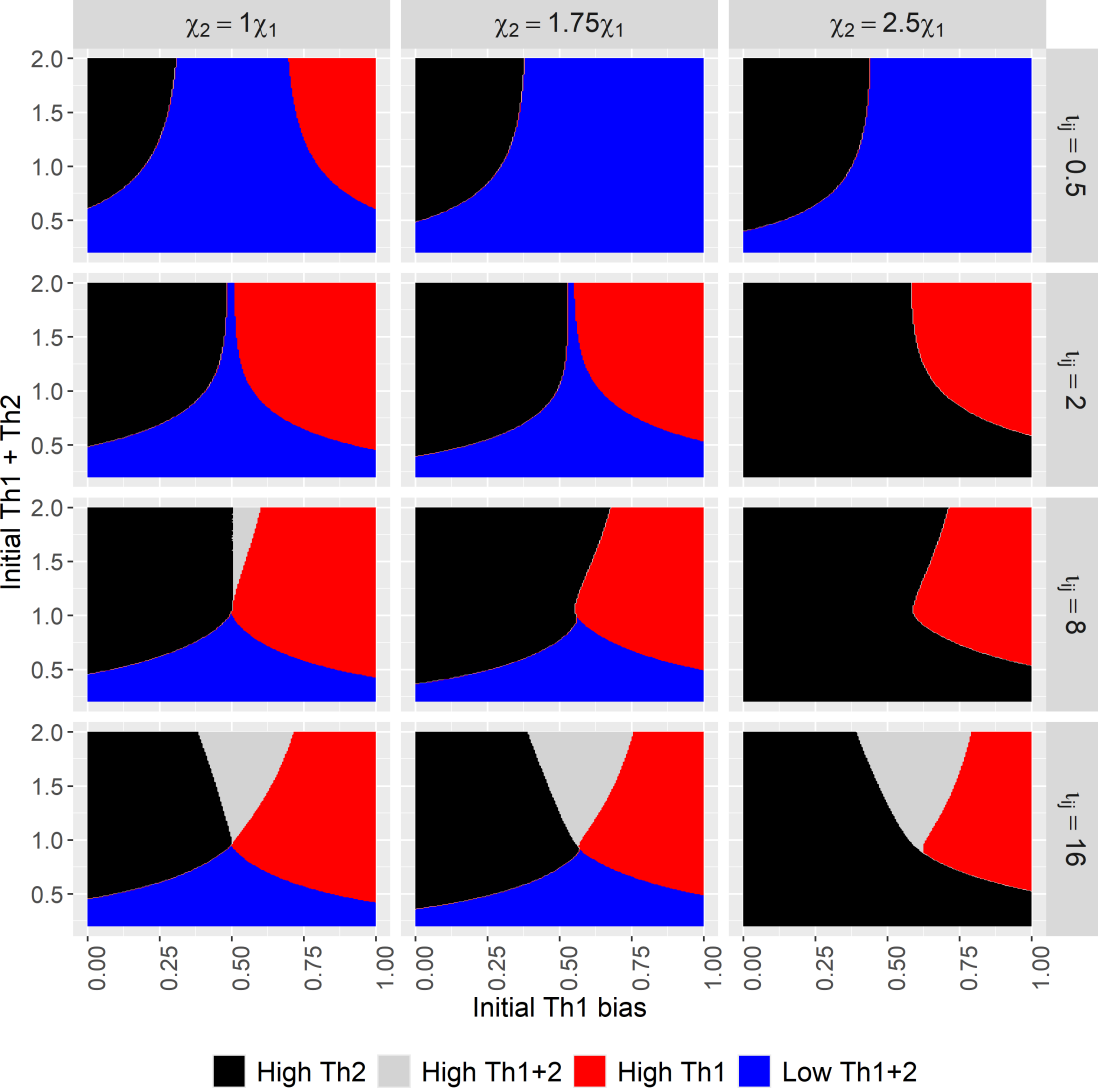
Infection outcomes as the initial immune state varies across values of $\chi_{1},\chi_{2}$ and $\iota_{12}=\iota_{21}$ . For the ‘high Th2’ outcome, the immune system is polarized towards a Th2 response and the parasite is extinct (acute infection). For the ‘high Th1+2’ outcome, there is a high level of both Th1 and Th2 immune cells and the parasite biomass is very low. For the ‘high Th1’ outcome, the immune system is polarized towards a Th1 response and parasite biomass is very high (chronic infection). For the ‘low Th1+2’ outcome, there is a low level of both Th1 and Th2 immune cells and the parasite biomass is high.

In the Th2-biased response case (figure 1, bottom row), we see that the basin of attraction for the Th2-polarized response is much larger initially, we never see the emergence of a high coactivation equilibrium, and at very high levels of activation, the polarized responses will be the only equilibria. This indicates that a growing parasite population that provokes a biased immune response can eventually lead to immune polarization. If the parasite is something like a gastrointestinal nematode, this polarization is far more likely to lead to clearance than chronicity (noting the much larger basin of attraction for the Th2-polarized response).

### (b) Incorporating parasite dynamics reveals dose-dependent effects

Although this model reveals that multistability is an intrinsic feature of the immune response, it ignores the reality that the immune response inhibits parasite growth; this may limit the possible outcomes, as negative feedbacks tend to be stabilizing [38]. To account for this, we extend the model to include negative feedbacks on parasite growth. We assume that the parasite is inhibited by a T-helper 2 response (e.g. it is an extracellular macroparasite), though that assumption is not critical to our results. We further assume that the parasite can directly stimulate both Th1 and Th2 responses according to the cytokine responses it induces in dendritic cells and other cells of the innate immune system; this reflects the biological reality that the detection and activation of T-cells by the parasite depend more upon antigen abundance and the contact rate between dendritic and T-cells than on the density of T-cells, *per se* [39]. We assume for simplicity that the parameters governing this response (the maximum activation rate $c_{i}$ and the half-saturation constant $C_{i}$ ) capture the dependence of this process on the abundances of antigen presenting cells and naïve T-cells, and the rate that these cells encounter one another [34]. Also, for simplicity, we assume a very simple form for the parasite dynamics: the parasite grows logistically in the absence of any immune regulation, and the parasite’s mortality rate is a linear function of the abundance of Th2 cells; in reality, of course, Th2 cells are not effector cells, so the per Th2-cell expulsion rate parameter, a , is assumed to capture all of the downstream activation of immune effectors triggered by the Th2 response. We note that P is best interpreted as parasite biomass and K as the maximum possible biomass since we are considering the dynamics of a macroparasite infection, and while macroparasites grow within their host, they typically do not multiply. However, identical functional forms, albeit with different interpretations, would work equally well to describe the dynamics of a replicating intracellular pathogen.


$$\frac{dT_{1}}{dt}=\underset{\begin{array}{c} External\;\\ activation \end{array}}{\underbrace{b_{1}}}+\underset{\begin{array}{c} Parasite\\ activation \end{array}}{\underbrace{\frac{c_{1}P}{C_{1}+P}}}\;+\underset{\begin{array}{c} Self-\\ activation \end{array}}{\underbrace{\left(\frac{s_{1}T_{1}^{p}}{S_{1}^{p}+T_{1}^{p}}\right)}}\underset{\begin{array}{c} Cross-\\ inhibition \end{array}}{\underbrace{\left(\frac{I_{12}^{q}}{I_{12}^{q}+T_{2}^{q}}\right)}}\;-\underset{Apoptosis}{\underbrace{mT_{1}}}$$

$$\frac{dT_{2}}{dt}=b_{2}+\frac{c_{2}P}{C_{2}+P}+\frac{s_{2}T_{2}^{p}}{S_{2}^{p}+T_{2}^{p}}\;\frac{I_{21}^{q}}{I_{21}^{q}+T_{1}^{q}}-mT_{2}$$

.$$\frac{dP}{dt}=\underset{Parasite\;growth}{\underbrace{rP\left(1-\frac{P}{K_{p}}\right)}}\;-\underset{Parasite\;expulsion}{\underbrace{aPT_{2}}}$$

We can again create a dimensionless version of this model. The dimensionless state variables corresponding to immunity and time are $t_{i}=T_{i}/S_{i}$ and τ=tm , as above, and a dimensionless measure of parasite biomass is $p=P/K_{p}$ , such that parasite biomass is measured relative to parasite carrying capacity. The dimensionless parameters of the model are $\beta_{i}=b_{i}/mS_{i}$ (baseline immune activation), $\sigma_{i}=s_{i}/mS_{i}$ (immune self-stimulation) and $\iota_{ij}=I_{ij}/S_{j}$ (immune cross-inhibition), as above. We introduce four new parameters: $\chi_{i}=c_{i}/mS_{i}$ (parasite-induced immune activation), $\kappa_{i}=C_{i}/K_{p}$ (sensitivity of immune activation to parasite biomass), ξ=r/m (parasite maximum growth rate),and $\alpha =aS_{2}/m$ (parasite loss per immune cell). Table 1 gives further interpretation of these new parameters. The dimensionless system is then:


$$\frac{dt_{i}}{d\tau }=\beta_{i}+\frac{\chi_{i}p}{\kappa_{i}+p}+\frac{\sigma_{i}t_{i}^{2}}{1+t_{i}^{2}}\frac{\iota_{ij}}{\iota_{ij}+t_{j}}-t_{i}\;\;\;for\;i,j=1,2$$

.$$\frac{dp}{d\tau }=\xi p\left(1-p\right)-\alpha t_{2}p$$

In figure 2, we show how infection outcomes depend on the initial immune state while holding the initial parasite biomass constant. Across the columns, we increase the bias in the parasite’s activation of Th2 cells over Th1 by simultaneously increasing $\chi_{2}$ and decreasing $\chi_{1}$ ; across the rows, we increase the relative sensitivity of self-activation to changes in the immune state by increasing both $\iota_{12}$ and $\iota_{21}$ . Because $\iota_{ij}=I_{ij}/S_{j}$ , increasing $\iota_{ij}$ implies increasing the value of $I_{ij}$ (the half-saturation constant for cross-inhibition) relative to $S_{j}$ (the half-saturation constant for self-activation). In the electronic supplementary material, we explore a much wider range of values for both $\chi_{i}$ and $\iota_{ij}$ as well as allow initial parasite biomass to vary.

Despite the negative feedback of a Th2 response upon macroparasite growth, we see the same four immune outcomes in figure 2 as we observed in figure 1: two equilibria representing polarization of the immune response, a high co-activation equilibrium, and a low co-activation equilibrium. These four states correspond to two qualitatively different outcomes, from the parasite perspective. When the immune system is polarized towards a Th2 response, the macroparasite population goes extinct, representing an acute infection. Similarly, when the immune system is at the high coactivation equilibrium, the parasite population is very close to extinction (when $\iota_{12}=\iota_{21}=8$ ) or is extinct (when $\iota_{12}=\iota_{21}=16$ ). When the immune system is polarized towards a Th1 response, the macroparasite reaches a high biomass, representing a chronic infection. Similarly, when the immune system is at the low co-activation equilibrium, the parasite population achieves high biomass.

We see a clear influence of the initial immune state on which equilibrium is reached. In particular, when the system starts biased towards Th2 (small values on the x-axis), Th2 polarization and an acute infection are more likely. Similarly, when the system starts biased towards Th1 (large values on the x-axis), Th1 polarization and a chronic infection are more likely. However, figure 2 also shows that the initial total immune abundance is also important, as the system is more likely to go towards the low co-activation equilibrium if it starts with a low abundance of T-helper cells of both types initially (small values on the y-axis).

It is also clear from this further development of the model that the possible equilibria depend on parameters governing the immune dynamics. For example, when cross-inhibition is more sensitive to changes in the immune state than self-activation ( $\iota_{12}=\iota_{21}=0.5$ ), the immune system is much more likely to end up in a low co-activation state that allows the parasite to flourish. The basin of attraction for this low co-activation state gets smaller, and the basin of attraction for the high co-activation equilibrium larger, as self-activation becomes more sensitive to changes in the immune state than cross-inhibition. As the parasite’s induction of immunity becomes increasingly Th2 biased, it becomes more likely that the system will end up at an equilibrium where the parasite is excluded.

**Figure 3.**
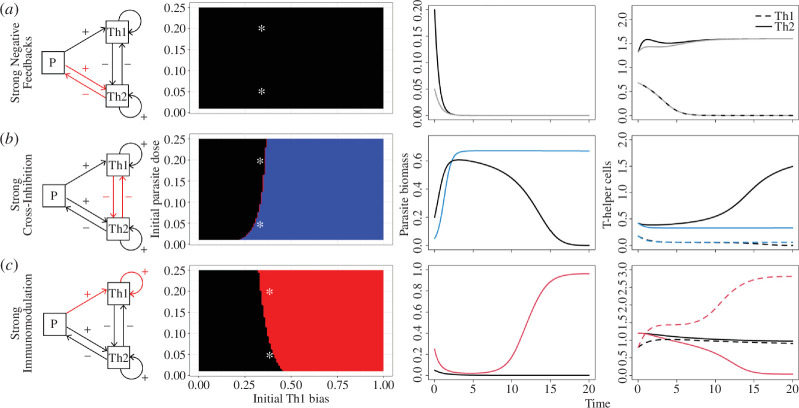
Variation in the infection outcome across initial parasite doses can reveal underlying immune mechanisms. (*a*) If the parasite activates a strong and Th2-biased immune response, it will be cleared (black area) regardless of the initial immune state or dose ( $\chi_{2}=1,\chi_{1}=0.04,\iota_{ij}=1$ ). Asterisks show the initial parameter values for the numerical simulation results shown in the rightmost columns. (*b*) If cross-inhibition is relatively strong, then even low doses of the parasite can lead to a chronic infection if initial Th1 bias is sufficiently high (blue area) whereas high doses are cleared across a wider range of initial Th1 biases (black area) ( $\chi_{2}=0.155,\chi_{1}=0.067,\iota_{ij}=8$ ). (*c*) If the parasite manipulates the immune system by activating the inappropriate Th1 response, then any dose can be cleared if initial Th1 bias is sufficiently low (black area) whereas high doses become chronic across a wider range of initial Th1 biases (red area) ( $\chi_{2}=0.04,\chi_{1}=1,\iota_{ij}=1)$ .

Initial parasite dose can also alter the infection outcome. For example, if the parasite activates the Th2 response much more than it activates the Th1 response ( $\chi_{2}\geq 3\chi_{1}$ ), then the parasite will be cleared regardless of dose (figure 3*a*). If cross-inhibition is relatively sensitive to changes in T-helper cell abundance (e.g., $\iota_{12}=\iota_{21}=1$ ) and the parasite stimulates Th2 immunity moderately more than Th1 (e.g. $\chi_{1}<\chi_{2}<3\chi_{1}$ ), then a low dose leads to low co-activation and a chronic infection, whereas a high parasite dose will lead to Th2 polarization and an acute infection (figure 3*b*). If the parasite is engaged in active immunomodulation (e.g. $\chi_{1}>\chi_{2}$ ), but self-activation is much more sensitive than cross-inhibition, then it is possible that low parasite doses can be cleared, but a high parasite dose will lead to Th1 polarization and an acute infection (figure 3*c*).

Notice that these dose responses can help reveal underlying details about the dynamics of the induced immune response, potentially providing mechanistic explanations for dose response variation observed in empirical studies [22–24]. In particular, if the infection is cleared regardless of dose (figure 3*a*), it indicates strong negative feedbacks: the parasite strongly stimulates the effective immune response, and that responses strongly impair parasite growth. On the other hand, if low doses lead to a chronic infection whereas high doses are cleared (figure 3*b*), it indicates that cross-inhibition is relatively sensitive to changes in the immune state and a low parasite dose will not stimulate Th2 immunity strongly enough to overcome this cross-inhibitory effect. Finally, if low doses lead to an acute infection whereas high doses become chronic (figure 3*c*), it indicates that the parasite is actively manipulating the immune response.

## 3. Discussion

Theoretical immunologists have long been aware that immune polarization, whether of T-cell populations or individual T-cell phenotypes, is driven by self-activating and cross-inhibiting feedback processes (e.g. Th1 cytokines increasing expression of the transcription factor T-bet, which regulates expression of Th1 cytokines [35,37]). However, this model is the first we are aware of that dynamically connects those immune feedbacks to a model of parasite growth. We show that (i) qualitatively distinct infection outcomes (clearance, and thus acute infection, versus chronicity) can result from changing the initial immune state and parasite dose and (ii) changing the strength of these feedbacks (e.g. by altering the values of the parameters $\iota_{12}$ and $\iota_{21}$ ) can alter the potential infection outcomes.

These feedback mechanisms are essential components of the immune response. Theoretical ecologists often analogize immune-parasite interactions with predator–prey interactions [40–42] but it is the self-activating and cross-inhibiting aspects of the immune response that reveal the limits of that analogy. Parasite clearance is only possible because of immune-driven positive feedback loops; in the absence of immune self-activation, immune activation would decrease alongside parasite abundance, leading to the coexistence of the immune system and parasite.

Indeed, recent theoretical studies [32,33] have shown how the inclusion of positive feedback loops can generate Allee effects that produce bistability between equilibria representing parasite clearance and chronic infection. Both of these models focused on positive feedbacks generated by parasite manipulation. In the model of van Leeuwen *et al*., the positive feedback loop is that the parasite growth increases the redirection of resources away from the immune response and towards parasite growth; thus, a low initial dose of parasite can be cleared but a high dose can become chronic. In the model of Ellner *et al*., the positive feedback loop is that the parasite growth increases inhibition of the immune response, producing the same pattern where a low dose is cleared but a high dose becomes chronic. And indeed, many parasites and pathogens do immunomodulate their hosts, either directly [43] or indirectly [44]. For example, the mouse whipworm, *Trichuris muris*, produces an excretory/secretory product that immunomodulates the host by binding to a key Th2 cytokine [45]; this inhibition causes the system to polarize towards an inappropriate Th1 response that is unable to clear the parasite.

Here, we show that immune thresholds that drive qualitative differences in the infection outcome can arise in the absence of parasite immunomodulation. The positive feedback loops and mutual inhibition loops inherent to the immune response can trigger incorrect immune polarization that in turn can flip the interaction from acute to chronic or vice versa. For example, in figure 2, even when the parasite’s activation of the Th2 response is 2.5 times stronger than its induction of the Th1 response, if the initial immune state is biased towards Th1, the self-activation and cross-inhibition feedbacks can nonetheless lead to Th1 polarization and a chronic infection. This reveals, lying within the feedback mechanisms of the immune response itself, the seeds of the immune system’s failure to clear a parasitic infection.

Our analysis also reveals nuance in infection outcomes beyond a simple acute/chronic dichotomy. In particular, we show that there are two immune phenotypes that can produce each outcome (figure 2). An acute infection can be produced by either Th2 polarization or high co-activation of both Th1 and Th2 (which is rarely observed empirically; see below), whereas a chronic infection is produced by either Th1 polarization or low co-activation of both Th1 and Th2. For helminth parasites, that Th2 polarization leads to clearance and Th1 polarization leads to a chronic infection is well established [25,26,46,47].

The low co-activation equilibrium is most common when cross-inhibition is more sensitive than self-activation (figure 2; $\iota_{12}=\iota_{21}=0.5$ ). In this case, the parasite is able to ‘fly below the radar’ of the immune response by never provoking activation strong enough to overcome the intrinsic tendency for cross-inhibition to lead to regulation. Some parasites, rather than inducing an inappropriate Th1 response to achieve chronic infection, do so by inducing a regulatory T-cell response that keeps both Th1 and Th2 immunity suppressed [48–50]. Our model suggests an alternative pathway towards a similar immune phenotype and resulting infection outcome.

The high co-activation equilibrium is most common when self-activation is much more sensitive than cross-inhibition (figure 2; $\iota_{12}=\iota_{21}=16$ ). It is tempting to suggest that this might be indicative of immunopathology, as some studies have shown that high levels of both Th1 and Th2 cytokines are correlated with immunopathology [51]. But immunopathology is more typically associated with either Th1 or Th2 polarization [52,53], and hybrid Th1/2 cells, which produce both Th1 and Th2 cytokines, are associated with reduced immunopathology [54]. Indeed, high co-expression of both Th1 and Th2 at the same time and place (e.g. lymph node or organ) is very rarely observed *in vivo*. This discrepancy between the model and data can be interpreted in at least two ways.

One interpretation relies on the fact that the stability of the high co-activation equilibrium requires that self-activation is far more sensitive to changes in the immune state than cross-inhibition (e.g. when $\iota_{ij}=16$ in figure 2). It is possible that this is simply a part of the parameter space that is unoccupied in nature; that hypothesis could be tested by confronting the model with data on the dynamics of immunity and parasite growth to estimate the parameters of the model [55]. An alternative interpretation is that the model is missing critical biology that is necessary to prevent the high co-activation equilibrium from emerging. In particular, our models are defined at the scale of the immune cell population, but cytokine signalling by individual T-cells emerges from the intracellular interaction between cytokine receptors and master transcription factors, such as Tbet and GATA3, such that it is individual T-helper cells that become polarized [35,37]. A previous model of the dynamics of cytokines and transcription factors predicts that the polarization of T-helper cell populations behaves like a quorum sensing process and is guaranteed to occur above a certain threshold cell population size [36]. That is, the production of cytokines by individual T-helper cells eventually produces a signal that is strong enough that it forces all the cells to adopt the same T-helper phenotype. Reasonably, therefore, if we were to extend our model to consider the dynamics of cytokine production and cellular signalling, we might eliminate the high co-activation equilibrium.

Our model also reveals that changing the parasite dose can also alter infection outcomes (figure 3). We show cases where a low dose can become chronic, whereas a high dose is cleared. In figure 3*b*, this is due to the changing dose shifting the infection outcome from low co-activation to Th2 polarization. In the electronic supplementary material, we also show cases where a low dose produces Th1 polarization, and a high dose produces Th2 polarization (electronic supplementary material, figure S3). In that case, this is due to an interaction between the parasite dose and the initial Th1-ness of the immune response. Regardless of the mechanism, this is the first model that is capable of producing this pattern of dose response, despite the fact that the pattern has been observed in multiple parasite species [25,26,56]. Our model is also capable of studying more complex parasite dose patterns. Here, we have assumed a single bolus infection, whereas in nature, hosts are commonly exposed to repeated low doses of parasites. Although rare, there are laboratory experiments that have contrasted the effects of bolus versus ‘trickle’ infections, showing that trickle infections can induce different immune phenotypes and infection durations [24,57]. For example, Bancroft *et al*. [24] found that mice could clear a bolus infection of 240 eggs of *Trichuris muris*, but would become chronically infected if those eggs were introduced in six doses of 40 eggs. Although we do not explore such experimental designs in detail here, we show in figure S12 of the electronic supplementary material that this model can accommodate more complicated dosing designs and, indeed, can recapitulate the results of Bancroft *et al*. [24], where a high dose of parasites is cleared by a polarized Th2 response, whereas a trickle dose of the same number of parasites becomes a chronic infection with no immune polarization.

Other models of the dose-dependence of induced immune responses and/or duration of infection have revealed further complexities (e.g. differential impacts of dose-dependence and time-dependence on induced immune responses [58] or impacts of constitutive responses on the dose-dependence of infection duration [59,60]), and we expect this to be a rich vein for further discoveries in within-host ecology.

More generally, our results suggest possible hypotheses for the variety of clearance phenotypes observed in nature. For example, many intracellular pathogens have a predictable and short infection duration, seldom, if ever, producing chronic infections. Our model suggests that this is caused by highly biased immune activation, coupled with moderate sensitivity of self-activation to changes in T-cell abundance (figure 3*a*; electronic supplementary material, figure S3–S5). High sensitivity can lead to incorrect polarization if the immune system starts out highly biased (figure 2; electronic supplementary material, S1,S2), whereas low sensitivity, relative to cross-inhibition, can lead to low co-activation (figure 2; electronic supplementary material, figure S6). Macroparasites such as gastrointestinal nematodes can often produce both acute and chronic infections, which our model suggests may be due to only moderate activation of immunity, coupled with moderate sensitivity of self-activation compared to cross-inhibition (figure 2).

These findings have a number of important implications for the interpretation of experimental data. Our model indicates that the initial immune state upon infection is critically important to the eventual duration of infection. In controlled experiments, for example with inbred mice, the initial immune state is often unmeasured. This is partly because of the limited sample types and volumes available longitudinally from small animals, and partly because inbred lines and conventional vivaria are designed to limit such variation; furthermore, researchers are often more focused on the effect of experimental treatment on the immunological outcome. Our results show that, even when the outcome would seem to be obvious (e.g. infection with a Th2-stimulating parasite producing Th2 polarization), the intrinsic feedback mechanisms within the immune system can drive the system towards an unexpected outcome (e.g. Th1 polarization), depending on the initial state of the system. This may help to explain the common experimental finding of variation in infection duration among hosts of a given genotype exposed to the same dose [15,61,62], even when hosts are exposed to clonal isolates of parasites such as malaria [63] or streptococci [64]. This suggests that experimental studies need to consider both the starting point and the endpoint of the immune system to make sense of the outcome of an experimental treatment.

Our results also have important implications for epidemiological studies. In particular, by modelling parasite transmission using compartmental models (e.g. SIR models [9]), epidemiologists are assuming that infection dynamics are, on average, the same across hosts. For example, the assumptions underlying a susceptible-infected-recovered (SIR) model are that all hosts can recover from infection and that there is an average recovery rate that is a good approximation of the distribution of recovery times (typically assumed to be exponential). Unfortunately, our model suggests that neither of these assumptions is likely to be met for many parasites: some individuals will become chronically infected, whereas others will recover quickly, producing a complex distribution of recovery times that cannot be easily captured by a simple compartmental framework. This will have important implications for both parasite transmission and evolution. For example, in a simple compartmental model that assumes a homogeneous host population, increasing recovery rate (decreasing duration) will lead to the evolution of higher virulence (assuming a classic virulence—transmission trade-off [65]). However, models that incorporate host heterogeneity in recovery have shown that this heterogeneity can lead to the evolution of lower or higher virulence, compared to the homogeneous case, depending on how evolution shifts the relative importance of different host types to parasite fitness [66–68]. Moreover, in these models, host heterogeneity is discrete and static, whereas our model suggests that the recovery rate of any individual will depend on its immunological history and on the dose of parasite it receives, implying that recovery rates become a function of epidemiology. Addressing that challenge likely necessitates multiscale models that can account for complex within-host immune–parasite interactions and between-host transmission [69,70].

Even with the complexity evidenced here, these models have left out a considerable amount of biological detail that may be important to consider. Whether a naïve T-helper cell becomes Th1 or Th2 depends on the intracellular interaction between cytokines and transcription factors [35–37]. These interactions are also governed by positive feedback loops, setting up the potential for intracellular multistability. Thus, a more biologically realistic model of immune polarization must also be multiscale, linking the within-cell and between-cell dynamics of cytokine expression and immune phenotype development. Additionally, the models presented here only consider immune activation, ignoring the dynamics of immune downregulation by regulatory T-cells. Such an extension would likely be critically important to understanding, for example, how these positive feedbacks affect the outcome of coinfection and sequential infection. Whether Th1 or Th2 polarization is likely to facilitate or inhibit a secondary infection depends critically on how long that polarization lasts, which itself may depend upon infection duration. This will also have implications at the population scale: because our results indicate that the initial immune state critically determines the outcome of infection, then whether a host will be susceptible to infection and whether it will clear that infection will depend on the host’s current immune state and its recent infection history.

More broadly, our model shows that immune–parasite interactions are subject to strong Allee effects [32,33]. Although Allee effects are thought to be important to population processes like extinction and invasion, documenting their existence is challenging, often due to limited experimental replication and difficulty measuring fitness [29]. Both of these limitations are overcome in immune–parasite interactions, as the scale of replication is the individual host, and parasite fitness is often relatively easy to measure. This suggests that parasites might be ideal systems for developing and testing methods for quantifying Allee effects. Moreover, the existence of Allee effects in immune–parasite systems might suggest novel strategies for combating infections that leverage the existence of persistence thresholds [30] to enhance host resistance against infection.

## Data Availability

Supplementary material is available online [71].
